# Behavioromics: A New Paradigm of Big Data-Powered Insights for Proactive Health

**DOI:** 10.34133/research.1205

**Published:** 2026-03-17

**Authors:** Jingman Shi, Junlong Li, Huanhuan Huang, Teng Teng, Hao Wu

**Affiliations:** ^1^ Center for Big Data and Intelligent Medicine, The First Affiliated Hospital of Chongqing Medical University, Chongqing 400016, P. R. China.; ^2^ Key Laboratory of Digital Health and Intelligent Medicine, Chongqing Municipal Health Commission, Chongqing 400016, P. R. China.; ^3^ Chongqing Translational Medicine Center, Chongqing 400016, P. R. China.; ^4^ Department of Scientific Research, The First Affiliated Hospital of Chongqing Medical University, Chongqing 400016, P. R. China.; ^5^ Department of Psychiatry, the First Affiliated Hospital of Chongqing Medical University, Key Laboratory of Major Brain Disease and Aging Research (Ministry of Education), Psychiatric Center of Chongqing Medical University the First Affiliated Hospital, Chongqing 400014, P. R. China.

## Abstract

Behavior plays a critical role in health and disease. Although molecular omics have advanced the understanding of biological mechanisms, they alone cannot explain the role of behavior in health, and traditional behavioral measurement technologies have struggled to capture the dynamic, multidimensional, and heterogeneous nature of real-world behaviors. With the development of digital technologies such as wearable devices, smartphone sensors, and ecological momentary assessment, behavioromics has emerged as a new research paradigm with a holistic systems perspective, integrating continuous, multimodal behavioral data with molecular, physiological, and environmental information. By organizing behavioral measurement, modeling, and interpretation within a unified analytical framework, behavioromics reveals dynamic behavioral patterns, supports mechanism-informed inference, and identifies actionable targets for early intervention. Despite ongoing challenges in data heterogeneity, causal interpretation, and ethical governance, behavioromics holds promise for reshaping disease prediction, early detection, and precision intervention, and for advancing proactive health through earlier, behavior-centered prevention.

As an old Chinese proverb states, “A hundred steps after a meal keep one healthy to ninety-nine”, underscoring the central role of behavior in health and disease. Although genomics, proteomics, and metabolomics have advanced understanding of molecular mechanisms, they do not capture the full spectrum of individual health, particularly the dynamic influence of behavior [1]. Prior research has typically examined single behavioral factors in relation to health risks [2]. However, real-world behavior is multidimensional, encompassing interrelated domains such as physical activity, sleep, diet, cognition, and emotion [3]. Moreover, behavior is heterogeneous, dynamic, and context-dependent, shaped by biological, environmental, and social determinants [4]. These features complicate precise measurement and analysis.

Advances in wearable devices, smartphone sensors, and ecological momentary assessment now enable continuous, high-resolution behavioral data collection. Combined with artificial intelligence (AI)-based analytics, these data support dynamic representation and pattern discovery, promoting systematic and multidimensional behavioral research [5]. A new paradigm is therefore needed to conceptualize behavior as an integrated construct within health science. Distinct yet complementary to digital phenotyping and computational behavioral science (focused on acquiring, representing, or predicting discrete behavioral signals), behavioral epidemiology (emphasizing population-level associations), and behavioral medicine (centered on behavior-informed clinical interventions), behavioromics conceptualizes behavior as an integrated, temporally evolving system that links cumulative spatiotemporal patterns with biological and physiological processes, thereby complementing molecular omics in explaining health and disease trajectories.

Accordingly, we define behavioromics as a systems-oriented framework that positions human behavior as an organized, dynamic component of health, integrating continuous behavioral patterns with biological and environmental contexts to enable mechanism-informed interpretation beyond descriptive associations.

## Toward a Systems Science of Behavioromics

**Fig. 1.**
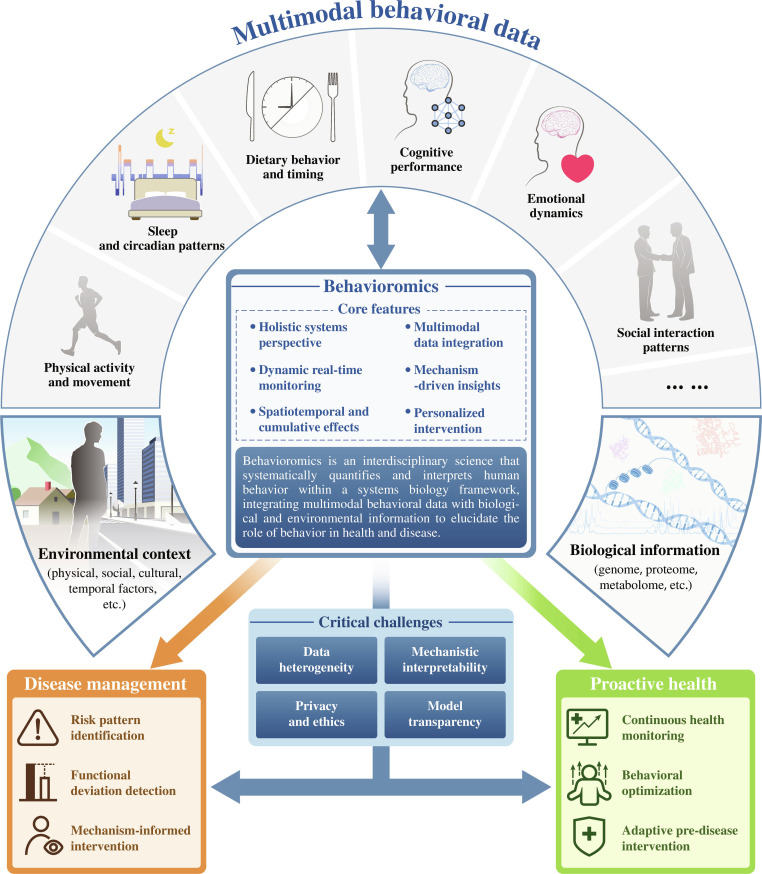
The framework of behavioromics.

Human behavior is a complex, responsive process through which individuals adapt to changes in internal and external environments, encompassing both muscle movements and mental acts. In behavioromics, these behavioral expressions are operationally distinguished into primary behavioral signals, which are directly and continuously measurable via digital devices (e.g., activity rhythms, sleep–wake cycles, mobility, and interaction traces), and higher-order behavioral states, which are computationally inferred from structured combinations of these signals (e.g., stress regulation, motivation, cognitive load, and affective dynamics) [6]. Drawing on the principles of “-omics” [7], behavioromics is an interdisciplinary science that systematically quantifies the patterns, structures, and dynamics of human behavior within a systems biology framework, integrating multimodal behavioral data with biological and environmental information to elucidate the role of behavior in health and disease (Fig. 1).

By enabling continuous monitoring and mechanism-informed interpretation of behavior—where observed behavioral patterns are interpreted in relation to underlying biological, physiological, or regulatory processes (e.g., linking irregular sleep-activity rhythms to circadian and autonomic dysregulation) rather than treated as isolated statistical associations—behavioromics supports proactive health, defined as the active management and optimization of health to prevent disease and maintain functional well-being. In this context, the systems biology framework refers to a system-level, mechanism-oriented perspective emphasizing interactions, feedback, and context-dependent responses, rather than specific experimental designs or fully specified mechanistic models.

## The Core Features of Behavioromics

Building on the framework, behavioromics is defined by 6 core features. Together, these features translate the systems-oriented framework into concrete analytical dimensions. First, a holistic systems perspective, integrating multiple dimensions and data sources to capture behavioral patterns in health and disease; second, dynamic real-time monitoring, which follows behavioral change over time instead of relying on static, cross-sectional measures; third, spatiotemporal and cumulative effects, which characterize behavior by its timing, frequency, intensity, duration, contextual location, and cumulative exposure over time; fourth, multimodal data integration, which combines behavioral, environmental, physiological, and molecular data from multiple sources and across multiple levels; fifth, mechanism-driven insights, which draw on methods such as machine learning, network analysis, causal inference, and reinforcement learning to identify behavioral clusters, feedback loops, and their associations with disease mechanisms; and sixth, personalized intervention, in which system-level insights support the design of individualized strategies for health promotion and disease prevention. Together, these features form a structured yet flexible framework for translating complex, real-world behavioral data into actionable insights for health research and practice, addressing long-standing limitations in behavioral research, including fragmented measurement, limited temporal resolution, weak connections to biological mechanisms, and insufficient support for proactive intervention design.

## Measurement and Analytical Foundations of Behavioromics

Behavioromics continuously collects multimodal behavioral data—physical activity, sleep, diet, cognition, emotions, and social interactions—using wearable sensors, mobile apps, environmental monitoring, and ecological momentary assessment [8]. These data can be integrated with molecular, physiological, and environmental information to map behaviors to underlying biological mechanisms. Ensuring accurate measurement requires strategies like time normalization, event-based alignment, interoperable standards, and standardized preprocessing [9].

To analyze complex behavioral data, researchers use machine learning, network science, causal inference, and multivariate modeling to quantify patterns, identify dynamic clusters, and characterize latent structures. These methods support complementary goals: risk prediction (e.g., pattern recognition and forecasting), mechanism discovery (e.g., network analysis and causal inference), and intervention optimization (e.g., reinforcement learning for adaptive strategies). By converting heterogeneous behavioral signals into computable, systematic representations, behavioromics enables mechanistic insights, predictive model development, and personalized behavioral interventions [10].

## Behavioromics in a Multidisciplinary Landscape

Behavioromics is inherently interdisciplinary, as human behavior reflects dynamic interactions among environmental, social, and physiological factors over time. Addressing this complexity requires an integrated analytical pipeline—from behavioral exposure definition and measurement to data integration, dynamic modeling, and biological interpretation—rather than parallel disciplinary efforts. This framework highlights coordination and cross-disciplinary tensions (e.g., tensions related to data sovereignty, measurement standards, and mismatches between epidemiological, computational, and biological interpretive frameworks) and structures the discipline-specific contributions discussed below [11].

Within behavioromics, epidemiology defines behavioral exposures across time, populations, and contexts, operationalizing them as time-varying variables aligned with health outcomes while addressing confounding, selection bias, and temporality [12]. These principles support causal inference but create tension, as traditional frameworks assume relatively stable exposures, whereas digitally measured behaviors are high-frequency and context-sensitive.

Sensor technologies translate these epidemiological constructs into continuous, real-world measurements, capturing primary behavioral signals such as activity rhythms, sleep–wake cycles, mobility patterns, and interaction traces. While this layer enables fine-grained behavioral dynamics, it also introduces tensions related to device heterogeneity, variable adherence, and context-dependent noise, which limit comparability and require careful downstream processing [8].

Software engineering ensures analytical coherence through synchronization, timestamp alignment, missing-data handling, and reproducible preprocessing pipelines [13]. Tensions emerge when proprietary systems limit interoperability or when real-time acquisition conflicts with reproducibility, forcing trade-offs between scalability and rigor.

Mathematical, statistical, and AI methods operate within design and measurement constraints, aiming not at unrestricted prediction but at extracting structured, interpretable patterns—such as temporal regularities, state transitions, and interaction networks—across populations and contexts [14]. A key tension lies in balancing model flexibility with transparency.

Systems biology links behavioral patterns to physiological regulation and molecular pathways [15]. By embedding behavioral signatures within multi-omics systems, behavioromics frames behavior as a modifiable component of health regulation rather than a descriptive correlate. Yet, the indirect observability of behavioral mechanisms requires theory-informed interpretation and cautious cross-scale inference.

## Applications of Behavioromics

Behavioromics supports disease management by identifying modifiable behavioral risk patterns and enabling mechanism-informed intervention. It is particularly relevant to chronic cardiometabolic diseases—including cardiovascular disease, diabetes, obesity, and metabolic syndrome—where behavior is central. By detecting subclinical risk trajectories, it shifts care from reactive treatment to prevention. At the population level, this approach may reduce healthcare burden and promote more equitable health management.

### Example 1: Disease management—Cardiometabolic disorders

In cardiometabolic disease management, contextual factors such as work schedules and meal availability shape behaviors including physical activity, sleep timing, and meal timing, which can be integrated with physiological signals from wearables and continuous glucose monitoring. Recurrent patterns—such as delayed meals combined with shortened sleep and reduced activity—are linked to biological dysregulation and increased cardiometabolic risk. Prospective evidence shows that unstable or delayed behavioral timing predicts higher diabetes risk: in the NutriNet-Santé cohort, first meals after 9:00 AM were associated with increased incident type 2 diabetes (hazard ratio [HR] = 1.59, 95% confidence interval [CI] 1.30 to 1.94) [16], and UK Biobank data indicate that greater sleep variability similarly elevates risk (HR = 1.59, 95% CI 1.33 to 1.90) [17]. These patterns reflect impaired insulin sensitivity, altered autonomic balance, and dysregulated postprandial glucose responses. By clarifying behavioral–physiological links, behavioromics supports targeted interventions, such as advancing meal timing to restore insulin–circadian alignment and prescribing time-specific activity to prevent disease progression.

### Example 2: Proactive health—Personalized health promotion

By integrating behavioral patterns with physiological feedback, including activity intensity, recovery indicators, and autonomic responses, individuals can make informed behavioral choices aligned with their functional state, preferences, and daily context. For example, exercise modality, intensity, and timing can be adaptively adjusted to enhance fitness and resilience while maintaining sustainable recovery. Such behavior-driven optimization supports self-regulation and the creation of supportive health environments. Because these interventions operate at the level of daily behavior adjustment rather than clinical treatment, they are inherently low-cost and scalable, reducing dependence on specialist care and enabling preventive support in populations with limited healthcare access.

## Critical Challenges and Future Directions in Behavioromics

Despite its potential for disease prediction and personalized intervention, translating behavioromics into practice faces several fundamental challenges.

First, behavioral data are heterogeneous and context-sensitive, originating from wearables, smartphones, and self-reports, each with varying sampling frequencies and reliability. Standardized data collection, preprocessing, and annotation frameworks are needed to enhance reproducibility and cross-cohort comparability.

Second, the causal pathways linking behavior to biological regulation and disease remain insufficiently defined. Without a clear mechanistic baseline, interpretability and clinical validation are constrained. Future research should prioritize multimodal causal inference and mechanism-oriented modeling to move beyond descriptive associations.

Third, computational models remain limited in transparency, generalizability, and trustworthiness. Many deep learning approaches may function as “black boxes” and are vulnerable to population bias and distribution shifts [18]. Advances in explainable AI, uncertainty-aware modeling, and cross-domain validation are essential for real-world implementation.

Large-scale behavioral data pose ethical and governance challenges due to sensitive emotional, behavioral, and social information. Key issues include data ownership, individual control, and algorithmic bias. Guidelines such as the World Health Organization’s guidance on *Ethics and Governance of Artificial Intelligence for Health* [19] emphasize fairness, transparency, and accountability, requiring behavioromics to comply. As monitoring shifts toward continuous, context-rich data, governance must move beyond static consent to protect individual agency while enabling responsible health use.

## Conclusion

Behavioromics is an emerging paradigm enabled by AI and digital measurement technologies, providing a systematic framework to quantify behavior and link it with physiological and biological states within environmental contexts. Through continuous monitoring and mechanism-informed interpretation, it supports dynamic understanding of health trajectories and advances proactive health management.

Its long-term impact depends on methodological rigor, interdisciplinary collaboration, and ethical implementation. By integrating behavioral and biological perspectives, behavioromics advances precision and proactive health, offering researchers, clinicians, and policymakers a shared foundation for earlier prevention, targeted intervention, and population-level planning based on real-world behavior. Future progress will require mechanism-aware behavioral representations that are robust across populations and contexts, enabling scalable translation of continuous data into actionable health insights.
